# Genome Sequence of the Mycotoxigenic Crop Pathogen *Fusarium proliferatum* Strain ITEM 2341 from Date Palm

**DOI:** 10.1128/MRA.00964-18

**Published:** 2018-09-06

**Authors:** Bandar F. Almiman, Taiwo A. Shittu, Sreenivasaprasad Muthumeenakshi, Riccardo Baroncelli, Surapareddy Sreenivasaprasad

**Affiliations:** a School of Life Sciences, Institute of Biomedical and Environmental Science and Technology (iBEST), University of Bedfordshire, Luton, United Kingdom; b University of Salamanca, Salamanca, Spain; Vanderbilt University

## Abstract

*Fusarium proliferatum* is a widely distributed fungal pathogen associated with more than 26 crop species important in global food security. Its strong mycotoxigenic capability with potential impacts on human and animal health is well recognized. In this work, we report the draft genome sequence of F. proliferatum strain ITEM 2341, originally isolated from date palm, providing a platform for further comparative and functional genomic investigations.

## Abstract (precis)

*Fusarium proliferatum* is a widely distributed fungal pathogen associated with more than 26 crop species important in global food security. Its strong mycotoxigenic capability with potential impacts on human and animal health is well recognized.

## ANNOUNCEMENT

*Fusarium proliferatum* is an emerging fungal pathogen belonging to the *F. fujikuroi* species complex within the *Fusarium* genus. The *F. fujikuroi* complex contains an estimated 50 species (1). F. proliferatum is widely distributed and associated with economically important crops such as rice, maize, sugarcane, date palm, onion, and tomato (2). F. proliferatum is able to cause different types of diseases, such as dieback, wilt, and rot, at different stages of the crop affecting various parts. F. proliferatum can also colonize diverse types of host plants, including maize and orchids, without displaying visible symptoms (3, 4). In addition to its direct impact on crop production, F. proliferatum is well recognized to produce high levels of mycotoxins, such as fumonisins, which are secondary metabolites known to be carcinogenic (4, 5). F. proliferatum ITEM 2341 was originally isolated in 1998 from the roots of date palm (*Phoenix dactylifera*) in Buraydah (Al-Qassim, Saudi Arabia; http://server.ispa.cnr.it/ITEM/Collection/).

The *F. proliferatum* strain ITEM 2341 was grown in potato dextrose broth for 5 days at 20°C. The mycelium was harvested using sterile muslin cheesecloth, air dried at room temperature for 15 min, and ground into a fine powder in liquid nitrogen with a sterile pestle and mortar. Genomic DNA was extracted from the mycelial powder using the GenElute plant genomic DNA miniprep kit (product number G2N350-1KT; Sigma, UK). The genome of F. proliferatum ITEM 2341 was sequenced using a combination of paired-end and mate pair libraries with the Illumina MiSeq platform using the service provider at the University of Cambridge, United Kingdom. Paired-end and mate pair libraries were prepared using the Illumina TruSeq PCR-free kit and the Illumina Nextera mate pair kit, respectively. The average length of DNA fragments used was 550 bp for paired-end sequencing and 2.5 to 4 kb for mate pair sequencing. The numbers of reads generated from the paired-end and mate pair libraries were 6,182,938 and 11,135,290, respectively. At 300 bases per read length, the total number of bases generated was ∼5.08 billion. FastQC version 0.11.5 was used to check the quality of the reads, and low-quality bases with a Phred score of less than Q20 as well as adaptor sequences were filtered using the BBDuk plugin within Geneious version 9.1.5. The curated reads were assembled using SPAdes version 3.5.0 (6), providing an approximate coverage of 111×.

The nuclear genome of *F. proliferatum* strain ITEM 2341 was assembled in 104 scaffolds with a total assembly size of 45.50 Mb (48.29% GC content). The genome assembly included the following parameters: an *N*_50_ value of 1,822,579 bp, an *L*_50_ value of 9, and a maximum scaffold size of 3,489,699 bases. Genome assembly quality assessment using BUSCO version 3.0.2 (7) with the Sordariomyceta data set (3,725 genes) showed the presence of 3,691 genes (99.1% gene content). The nuclear genome was annotated using the MAKER2 pipeline (8), and 14,379 protein-coding gene models were predicted. SignalP version 4.1 (9) analysis revealed 1,488 secreted proteins (10.35% of the predicted proteome). All software used for the assembly and analysis of the genome was set at default parameters.

The genome sequence of F. proliferatum ITEM 2341 represents a useful platform for further comparative genomic analysis of the adaptive divergence in this emerging pathogen (10–12).

### Data availability.

The whole-genome shotgun project has been deposited in DDBJ/ENA/GenBank under the accession number PKMI00000000 (BioSample number SAMN08122925; BioProject number PRJNA420865; NCBI Sequence Read Archive [SRA] accession numbers SRX4488677 and SRX4488678 [raw reads]) and released for access by the research community. The version described in this paper is PKMI01000000.
